# Antibiotic prophylaxis in transurethral resection of bladder tumours: study protocol for a systematic review and meta-analysis

**DOI:** 10.1186/s13643-020-01353-2

**Published:** 2020-04-23

**Authors:** Kathrin Bausch, Soheila Aghlmandi, Sarah Ursula Sutter, Linda Maria Stamm, Hannah Ewald, Christian Appenzeller-Herzog, Jan Adam Roth, Andreas F. Widmer, Hans-Helge Seifert

**Affiliations:** 1 Department of Urology, University Hospital Basel, Spitalstrasse 21, 4031 Basel, Switzerland; 2 University of Basel, Basel, Switzerland; 3 Basel Institute for Clinical Epidemiology and Biostatistics, University Hospital Basel, Spitalstrasse 12, 4056 Basel, Switzerland; 4 University Medical Library, University of Basel, Spiegelgasse 5, 4051 Basel, Switzerland; 5 Division of Infectious Diseases and Hospital Epidemiology, University Hospital Basel, Petersgraben 31, 4031 Basel, Switzerland

**Keywords:** Antibiotics, Antibiotic prophylaxis, Antibiotic resistance, Antimicrobial stewardship, Bacteriuria, Endourological surgery, Guideline, Recommendation, Transitional cell carcinoma, Urothelium cell carcinoma

## Abstract

**Background:**

The necessity of antibiotic prophylaxis for postoperative urinary tract infections (UTIs) after transurethral resection of bladder tumours is controversial. This potentially leads to the overuse of antibiotic prophylaxis and rising antimicrobial resistance rates. The objective of this systematic review and meta-analysis is to compare the impact of different antimicrobial prophylaxis schemes versus placebo on the prevention of postoperative UTI and asymptomatic bacteriuria.

**Methods:**

We designed and registered a study protocol for a systematic review and meta-analysis of randomized controlled trials and non-randomized (e.g. cohort, case-control) studies examining any form of antibiotic prophylaxis in patients with transurethral resection of bladder tumours. Literature searches will be conducted in several electronic databases (from inception onwards), including MEDLINE (Ovid), EMBASE (Ovid), and the Cochrane Central Register of Controlled Trials (CENTRAL). Grey literature will be identified through searching conference abstracts. The primary outcome will be postoperative urinary tract infections. The secondary outcome will be asymptomatic bacteriuria. Two reviewers will independently screen all citations, full-text articles, and abstract data. Potential conflicts will be resolved through discussion. The study methodological quality (or bias) will be appraised using appropriate tools (e.g. Risk of Bias 2.0 tool and Newcastle-Ottawa Scale). If feasible, we will conduct random-effects meta-analysis of outcome data. Additional analyses will be conducted to explore the potential sources of heterogeneity (e.g. study design, publication year, the setting of the study, and antibiotics regimen).

We will also search, identify, and discuss potential risk factors for urinary tract infections following transurethral resection of bladder tumours. This may serve as basis for a scoping review.

**Discussion:**

In times of rising antimicrobial resistance rates, sound evidence on the necessity of antibiotic prophylaxis is essential for implementation into guideline recommendations and for decision-making in clinical practice.

**Systematic review registration:**

PROSPERO, CRD42019131733

## Background

Bladder cancer is the most common malignancy of the male and female urinary tract system [1]. For primary diagnostics of bladder cancer, international guidelines recommend transurethral resection of suspicious lesions to obtain histology for a final exclusion of malignancy and as a treatment option for non-muscle invasive bladder cancer [2]. According to the World Health Organization, transurethral resection of bladder tumours (TURB) is classified as a clean-contaminated wound procedure [3]. In general, in clean-contaminated procedures, perioperative antibiotic prophylaxis (AP) is recommended to prevent postoperative urinary tract infections (UTIs) [4].

Nevertheless, in contrast to transurethral resection of the prostate (TURP) [5, 6], UTI rates are lower following TURB, and there appears to be little evidence for any benefit of AP. A lack of consensus was demonstrated in a recently published review that investigated the guidelines of the American Urological Association (AUA) and the Canadian (CAU), European (EAU), and Japanese (JAU) Associations of Urology [7]: The AUA considers TURB along with TURP and consequently AP should be applied to all patients, whereas the CAU groups TURB with cystoscopy and recommends AP only in high-risk patients. JAU guidelines recommend AP for TURB based on data for other transurethral procedures but state that low-risk cases without preoperative UTI can be considered to require no AP. EAU guidelines suggest assessing risk factors for postoperative infections to determine the use of AP. However, a closer definition of these risk factors is missing in the EAU guidelines.

The EAU recommendations are mainly based on one review of randomized controlled trials (RCTs), which included only two studies (*n* = 152) that compared AP to placebo [8]. Both studies are from the 1980s and found a nonsignificant decrease in the incidence of post-TURB bacteriuria with the use of AP compared to placebo (24.1% vs. 9.4% and 17% vs. 4.5%) and a 0% incidence of symptomatic UTIs in both groups. It was concluded that there is moderate- to low-grade evidence suggesting that AP is not necessary for TURB [8].

The heterogeneity in the recommendations underline that there is no consensus regarding the use of AP in the prevention of UTI after TURB in international guidelines. Nonetheless, application of AP after TURB should be carefully considered as unnecessary antibiotics increase the risk for emergence of multiresistant pathogens. Another complication comes from the lack of information in the guidelines on risk factors for UTI after TURB, which challenges the implementation of recommendations given for, e.g. “high-risk patients” only. Furthermore, while risk factors for postoperative UTI after transurethral surgery in general have been identified (see e.g. [9]), risk factors for UTI specifically after TURB have so far not been put together in a comprehensive way.

Taken together, the level of evidence for the necessity of AP for postoperative UTIs following TURB remains insufficiently documented. Moreover, even though consideration of risk factors is recommended, only little is known about risk factors for UTI after TURB.

The aims of this systematic review and meta-analysis are to compare the impact of AP schemes vs. placebo in patients undergoing TURB with the primary outcome of symptomatic UTI and asymptomatic bacteriuria (ABU) secondary.

Beyond the systematic review, we will explore and describe potential risk factors for UTIs following TURB as basis for future research.

## Methods

The present protocol has been registered within the PROSPERO database (registration number CRD42019131733) and is reported in accordance with the reporting guidance provided in the “Preferred Reporting Items for Systematic Reviews and Meta-Analyses Protocols (PRISMA-P)” statement (Additional file 1: Table S1) [10]. The planned systematic review and meta-analysis will be conducted in accordance with the “Centre for Reviews and Dissemination (CRD)” guidelines [11].

### Eligibility criteria

Studies will be included according to the following criteria: participants, study design(s), intervention, comparators and outcome(s) of interest. These criteria are: Participants: We will include studies investigating patients ≥18 years (regardless of sex and age) undergoing TURB. Study design: Eligible studies will be RCTs and non-randomized (e.g. observational) studies, including cohort studies and case-control studies. We will exclude case reports, case series, and single-arm cohort studies. Interventions and comparators: We will include any scheme of AP (i.e. any duration varying from a single-dose AP to several days, any oral or intravenous antibiotic substance eligible for the treatment or prophylaxis of UTI) vs. placebo (or no AP) and that report at least one pertinent outcome in patients undergoing TURB. Non-antibiotic prophylactic schemes are not eligible (e.g. bladder instillations with antibiotics or hyaluronic acid/chondroitin sulphate, D-mannose, immunoactive prophylaxis). Outcomes of interest: The primary outcome will be postoperative symptomatic UTI defined as symptoms including flank pain, urgency, frequency, suprapubic pain, fever, and antimicrobial confirmation of bacteriuria (≥ 10^5^ colony-forming units) according to the definition of the Centers for Disease Control and Prevention or as clinical diagnosis of cystitis, pyelonephritis, prostatitis, and urosepsis. The secondary outcome will be ABU (≥ 10^5^ colony-forming units). The outcomes have to occur until 60 days following the intervention.

For the exploration of potential risk factors for UTIs following TURB, we will include any studies (RCTs and non-randomized (e.g. observational) studies) that investigate risk factors for UTIs (defined as described above) in patients ≥ 18 years (regardless of sex and age) undergoing TURB independent of the AP scheme. Examples of risk factors for UTIs after TURB, which we anticipate to find based on the knowledge from studies on risk factors in lower urinary tract surgeries are health status, duration of catheterization, and duration of operation [9].

### Search strategy for identifying relevant studies

Two information specialists (H.E. and C.A.-H.) will develop the search strategies. The strategy for the systematic review (referred to as the “systematic search”) is concerned with AP in TURB. Text words (synonyms and word variations) and database-specific subject headings for TURB and AP will be used. We will search the electronic databases MEDLINE (Ovid) Embase (Ovid), and the Cochrane Central Register of Controlled (CENTRAL) from inception onwards (see draft search in Additional file 2).

The strategy for the exploration of potential risk factors (referred to as “exploratory search”) will be concerned with the identification and gathering of risk factors for UTI after TURB. It will be based on free-text synonyms and MeSH terms for TURB and UTIs and conducted in MEDLINE (see draft search in Additional file 3). To find additional references, we will also look out for potential risk factors while screening the results of the systematic search. The following supplementary search techniques apply to both the systematic review and the risk factors.

To identify additional conference abstracts, the abstracts of the Annual EAU Congresses 2016–2019 will be hand-searched in the European Urology Supplements on ScienceDirect. To identify possible additional studies that escape our electronic database searches, we will screen the bibliographic references of all included articles as well as the citations of those that are indexed in Scopus or the Web of Science.

### Selection of studies for inclusion in the review

All retrieved references will be exported to Endnote X9 (Endnote Version X9.1.1, Clarivate Analytics, 2019). Duplicates will be removed using EndNote X9. Two reviewers (K.B. and L.M.S.) will screen the references based on their titles and abstracts. All potentially relevant references will be retrieved in full-text and independently assessed by two reviewers (K.B. and L.M.S.). Any disagreements over eligibility will be resolved by consensus. Where necessary, a third review author (H.-H. S.) will make a final judgement. This applies to both the systematic and the exploratory search.

### Risk of bias assessment

The Risk of Bias 2.0 (RoB.2.0) tool will be used to assess the risk of bias in RCTs [12] and the Newcastle-Ottawa Scale for non-randomized studies [13] included in the systematic review. Assessments will be carried out independently by two reviewers (K.B. and S.U.S.) at study-level, with discrepancies being discussed with a third reviewer (H.-H.S.) to reach a consensus.

We will not assess risk of bias for studies that are only included for the exploration of potential risk factors.

### Data extraction and management

Data for the systematic review will be extracted and entered into a pre-defined and piloted Microsoft Excel database (Version 16.23, Microsoft Excel, 2019). Data will be extracted by one reviewer (K.B.) and independently checked by a second reviewer (L.M.S.). Discrepancies will be identified and resolved through discussion (with a third author where necessary, S.U.S.). Data items to be extracted will include the name of the first author, year of publication, study location, setting, and study design. Furthermore, the number of participants in each group, participant demographics (e.g. age, gender), any reported confounding factors, definition of AP (e.g. regime, duration), control intervention (i.e. including placebo, no treatment, standard of care, and other active treatments), definition of UTI, number of symptomatic UTI or ABU events per group, assessment of risk factors for UTI, and duration of follow-up are information required for risk of bias assessments.

For the exploration of risk factors, we aim to extract any reported risk factors for UTI after TURB as well as the respective statistical methods that were used to identify them.

### Data synthesis

We will perform a meta-analysis to assess the impact of AP on symptomatic UTI as a primary outcome. Using the data extracted from each paper (e.g., study design, design characteristics, author, year, quality of the study), we will build the evidence table(s) of an overall description of included studies to explore possible heterogeneity descriptively. The random-effects model will be used then to account for potential statistical heterogeneity, reporting the suitable effect measure and the respective 95% confidence interval (CI). The choice of a weighting method for pooling the results of the studies will account for the rare events setting. This is because the baseline risk for UTI is usually < 5% [9]; therefore, we expect that studies included in the meta-analysis might have a low number of events. Meta-analyses of studies with rare events face challenges that should be tackled using suitable statistical methods and proper effect measure(s) [14]. Either odds ratio (OR) or relative risk (RR) with their respective 95% CI will be reported depending on what authors used in the original publications. However, if we observe a high number of studies with a low number of events and/or no events, we will use OR because in rare events settings, OR and RR are almost the same, but OR has some computational advantages [15].

The effect measure of interest will be computed using the *metabin* function of the *rmeta* package [14]. Forest plot(s) will be generated to show the individual and pooled effect measure, 95% CI, the author’s name, publication year, and study weights for primary studies.

Heterogeneity between the results of the primary studies will be assessed using the Cochran’s *Q* test and will be quantified with the *I*-squared statistic. Assessment of the heterogeneity form the *I*-square will be considered low, moderate, or high when the values are below 25%, between 25% and 75%, or above 75%, respectively [16].

Sources of inter-study heterogeneity will be investigated with the random-effects meta-regression analysis that will be based on the following primary study characteristics: study design, publication year, the setting of the study, and antibiotics regimen. The meta-regression analysis will be weighted to account for both within-study variances of treatment effects and the residual between-study heterogeneity.

Meta-analysis will also be conducted for the secondary outcome ABU (≥ 10^5^ colony-forming units) using the appropriate effect measures and the methods proposed above.

As we do not know much about the number of events for the outcomes (primary and secondary), we might have to adjust the meta-analysis methods proposed above. The analyses will be carried out using the R statistical software (R version 3.4.4, R. Foundation for Statistical Computing, Vienna, Austria, 2018).

For the risk factors, we will check the statistical methods used to identify the risk factors for UTI and will report a list of the most important risk factors for UTI descriptively.

### Meta-biases

For the results of the meta-analysis, publication bias will be assessed using a funnel plot (a plot of effect estimates against sample sizes). A symmetrical shape of the graph will be interpreted as absence of publication bias, whereas an asymmetrical shape of the graph will be interpreted as presence of publication bias [17].

We will not assess publication bias for the exploration of the risk factors.

## Discussion

Antimicrobial resistance is particularly prevalent among the main pathogens of the urogenital tract [18]. Pathogens from urological patients show high antimicrobial resistance rates [19]. This has been explained by an overuse and a frequently extended use of antibiotic agents partly for AP in standard urological procedures [20].

Thus, guidelines should ensure that the use of AP is carefully considered and that AP is reduced to a minimum without increasing the postoperative complications for individual patients.

Bladder cancer is the most common malignancy of the male and female urinary system [1], and TURB is the most frequently performed procedure in diagnosis and treatment of bladder cancer [2]. However, guideline recommendations on AP in TURB rely on outdated and underpowered trials [8]. This leads to a low level of evidence and overt discrepancies in international guideline recommendations ranging from complete AP omission to complete AP application for several days [7].

Moreover, the rising antimicrobial resistance rates today impose a reasonably dosed use of AP and the careful evaluation of risk factors for postoperative UTI [9].

Thus, an up-to-date systematic review with meta-analysis may be able to provide reliable evidence to guide decision-making on whether or not to use AP. Furthermore, the collated knowledge on risk factors for UTI following TURB will inform future studies to assess which patients would benefit from AP. Such knowledge can help to reduce the use of AP to a necessary minimum.

Overall, carefully considered AP use may reduce the risk for UTIs and minimize the development of antibiotic-resistant pathogens. Furthermore, any measure to decrease antibiotic resistance rates has important beneficial consequences for public health in general.

### Strengths and limitations

Only a systematic review and meta-analysis would be able to provide reliable evidence to guide decision-making on whether AP can be recommended in this setting or not.

A strength of our study is the comprehensive search in three major databases, citation chasing on included records and hand-searching of conference contributions in order to comprehensively retrieve potentially relevant records. Limitations include the expected heterogeneity in the sample size of the retrieved studies, quality of the study design, rare outcome events, and definition and assessment of the primary outcome. Voiding symptoms such as dysuria or suprapubic pain can be caused by the TURB itself and the catheterization and might not only be a sign of UTI. Therefore, we decided to choose a combined outcome definition of symptoms and bacteriuria according to the definition of the Centers for Disease Control and Prevention.

In addition to the systematic review, we would like to get an impression of potential risk factors for UTI following TURB. As this investigation is merely explorative and not intended as a systematic overview, the search is intentionally less comprehensive as it would be for a systematic review. This means that any results shall only serve as rationale for conducting a full systematic scoping review in the future and not to inform clinical decision-making in any kind.

Any deviations from the protocol will be outlined in the final publication.

## Supplementary information

**Additional file 1.**

**Additional file 2.**

**Additional file 3.**

## Data Availability

Both systematic search strategy and exploratory search strategy can be found in the Additional file 1. The datasets, which will be used and analysed during the current study, are available from the corresponding author on reasonable request after completion of the analysis.

## Abbreviations

ABU: Asymptomatic bacteriuria
AP: Antibiotic prophylaxis
AUA: American Urological Association
CAU: Canadian Association of Urology
CENTRAL: Cochrane Central Register of Controlled Trials
CRD: Centre for Reviews and Dissemination
EAU: European Association of Urology
JAU: Japanese Association of Urology
OR: Odds ratio
PROSPERO: International Prospective Register of Systematic Reviews
PRISMA-P: Preferred reporting items for systematic reviews and meta-analyses for protocol
RCT: Randomized controlled trials
Rob.2.0: Risk of bias.2.0 tool
RR: Relative risk
TURB: Transurethral resection of bladder tumours
TURP: Transurethral resection of the prostate
UTI: Urinary tract infection
