# Supplementary material for: Antibiotic prophylaxis in transurethral resection of bladder tumours: study protocol for a systematic review and meta-analysis
Source: Syst Rev. 2020 Apr 23;9:89. doi: 10.1186/s13643-020-01353-2 (PMC7181504; doi:10.1186/s13643-020-01353-2)
Supplement: Supplementary file 2 — Additional file 2. [file 13643_2020_1353_MOESM2_ESM.docx]

# Additional file 2: Search strategy for AP in TURB

## Embase.com

((('transurethral resection'/de OR 'urologic surgery'/de OR 'urinary tract surgery'/de OR 'urethra surgery'/de OR (((transurethra* OR trans-urethra* OR intravesical OR vesical OR endovesical OR endoscopic OR ‘outflow tract’) NEAR/3 (surger* OR resection* OR operation* OR procedure* OR surgical)) OR electroresection OR electro-resection OR cauterization):ab,ti) AND ('transitional cell carcinoma'/de OR 'urothelial bladder cancer'/de OR 'bladder disease'/exp OR 'bladder dysfunction'/de OR 'bladder injury'/de OR 'bladder'/exp OR 'urothelium'/de OR bladder:ab,ti OR ‘vesica urinaria’:ab,ti OR intravesical:ab,ti OR vesical:ab,ti OR urotheli*:ab,ti OR ‘urinary tract epitheli*’:ab,ti OR uroepitheli*:ab,ti)) OR TURBT:ab,ti OR TURB:ab,ti)

AND ('Antibiotic Prophylaxis'/exp OR 'infection prevention'/de OR 'infection control'/de OR (('antiinfective agent'/de OR 'antibiotic agent'/exp OR 'antimicrobial therapy'/de OR 'antibiotic therapy'/de OR 'beta lactamase inhibitor'/exp OR 'urinary tract antiinfective agent'/de OR antibiotic:ab,ti OR antibiotics:ab,ti OR anti-biotic:ab,ti OR anti-biotics:ab,ti OR anti-bacterial:ab,ti OR antibacterial:ab,ti OR bacteriocidal:ab,ti OR bacteriocide:ab,ti OR bacteriocides:ab,ti OR Antiinfective:ab,ti OR Anti-infective:ab,ti OR antiseptic:ab,ti OR antiseptics:ab,ti OR anti-septic:ab,ti OR anti-septics:ab,ti OR antimicrobial:ab,ti OR anti-microbial:ab,ti OR microbicide:ab,ti OR microbicides:ab,ti OR infection*:ab,ti OR ‘biapenem’:ab,ti OR ‘brobactam’:ab,ti OR ‘carbapenem’:ab,ti OR ‘clavulanate potassium’:ab,ti OR ‘clavulanic acid’:ab,ti OR ‘doripenem’:ab,ti OR ‘ertapenem’:ab,ti OR ‘fropenem’:ab,ti OR ‘imipenem’:ab,ti OR ‘l 786392’:ab,ti OR ‘lenapenem’:ab,ti OR ‘meropenem’:ab,ti OR ‘monobactam ‘:ab,ti OR ‘nacubactam’:ab,ti OR ‘nocardicin’:ab,ti OR ‘nocardicinic acid ‘:ab,ti OR ‘panipenem’:ab,ti OR ‘pirazmonam’:ab,ti OR ‘razupenem’:ab,ti OR ‘ritipenem’:ab,ti OR ‘sanfetrinem’:ab,ti OR ‘sulbactam’:ab,ti OR ‘sulopenem’:ab,ti OR ‘sultamicillin’:ab,ti OR ‘tazobactam’:ab,ti OR ‘tebipenem’:ab,ti OR ‘thienamycin’:ab,ti OR ‘timentin’:ab,ti OR ‘tomopenem’:ab,ti OR ‘tribactam’:ab,ti OR ‘trinem derivative’:ab,ti OR ‘u 78608’:ab,ti OR ‘benzathine cefalexin’:ab,ti OR ‘benzathine cefapirin’:ab,ti OR ‘carbacephem ‘:ab,ti OR ‘cefacetrile’:ab,ti OR ‘cefaclor’:ab,ti OR ‘cefadroxil’:ab,ti OR ‘cefalexin’:ab,ti OR ‘cefaloglycin’:ab,ti OR ‘cefaloram’:ab,ti OR ‘cefaloridine’:ab,ti OR ‘cefalotin’:ab,ti OR ‘cefamandole’:ab,ti OR ‘cefapirin’:ab,ti OR ‘cefatrizine’:ab,ti OR ‘cefazaflur’:ab,ti OR ‘cefazedone’:ab,ti OR ‘cefazolin’:ab,ti OR ‘cefbuperazone’:ab,ti OR ‘cefcanel’:ab,ti OR ‘cefcapene’:ab,ti OR ‘cefclidin’:ab,ti OR ‘cefdaloxime’:ab,ti OR ‘cefdinir’:ab,ti OR ‘cefditoren’:ab,ti OR ‘cefepime’:ab,ti OR ‘cefetamet’:ab,ti OR ‘cefetecol’:ab,ti OR ‘cefiderocol’:ab,ti OR ‘cefixime’:ab,ti OR ‘cefluprenam’:ab,ti OR ‘cefmatilen’:ab,ti OR ‘cefmenoxime’:ab,ti OR ‘cefmetazole’:ab,ti OR ‘cefminox’:ab,ti OR ‘cefodizime’:ab,ti OR ‘cefonicid’:ab,ti OR ‘cefoperazone’:ab,ti OR ‘ceforanide’:ab,ti OR ‘cefoselis’:ab,ti OR ‘cefotaxime’:ab,ti OR ‘cefotetan’:ab,ti OR ‘cefotiam’:ab,ti OR ‘cefovecin’:ab,ti OR ‘cefoxitin’:ab,ti OR ‘cefozopran’:ab,ti OR ‘cefpimizole’:ab,ti OR ‘cefpiramide’:ab,ti OR ‘cefpirome’:ab,ti OR ‘cefpodoxime’:ab,ti OR ‘cefprozil’:ab,ti OR ‘cefquinome’:ab,ti OR ‘cefradine’:ab,ti OR ‘cefroxadine’:ab,ti OR ‘cefsulodin’:ab,ti OR ‘ceftaroline’:ab,ti OR ‘ceftazidime’:ab,ti OR ‘cefteram’:ab,ti OR ‘ceftezole’:ab,ti OR ‘ceftibuten’:ab,ti OR ‘ceftiofur’:ab,ti OR ‘ceftizoxime’:ab,ti OR ‘ceftobiprole’:ab,ti OR ‘ceftolozane’:ab,ti OR ‘ceftriaxone’:ab,ti OR ‘cefuroxime’:ab,ti OR ‘cefuzonam’:ab,ti OR ‘cephalosporin*’:ab,ti OR ‘cephamycin’:ab,ti OR ‘deacetoxycephalosporin C’:ab,ti OR ‘deacetylcefotaxime’:ab,ti OR ‘deacetylcephalosporin C’:ab,ti OR ‘fleroxacin deacetylcefotaxime ester’:ab,ti OR ‘flomoxef’:ab,ti OR ‘latamoxef’:ab,ti OR ‘loracarbef’:ab,ti OR ‘nitrocefin’:ab,ti OR ‘thiophenoxycefalotin’:ab,ti OR ‘aztreonam’:ab,ti OR ‘carumonam’:ab,ti OR ‘gloximonam’:ab,ti OR ‘sulfazecin’:ab,ti OR ‘tigemonam’:ab,ti OR ‘adicillin’:ab,ti OR ‘almecillin’:ab,ti OR ‘aminopenicillin’:ab,ti OR ‘amoxicillin’:ab,ti OR ‘ampicillin’:ab,ti OR ‘apalcillin’:ab,ti OR ‘aspoxicillin’:ab,ti OR ‘azidocillin’:ab,ti OR ‘azlocillin’:ab,ti OR ‘bacampicillin’:ab,ti OR ‘bacmecillinam’:ab,ti OR ‘carbenicillin’:ab,ti OR ‘carfecillin’:ab,ti OR ‘carindacillin’:ab,ti OR ‘cloxacillin’:ab,ti OR ‘cyclacillin’:ab,ti OR ‘dicloxacillin’:ab,ti OR ‘epicillin’:ab,ti OR ‘flucloxacillin’:ab,ti OR ‘flumoxil’:ab,ti OR ‘fomidacillin’:ab,ti OR ‘furbenicillin’:ab,ti OR ‘fuzlocillin’:ab,ti OR ‘hetacillin’:ab,ti OR ‘isopenicillin N’:ab,ti OR ‘lenampicillin’:ab,ti OR ‘mecillinam’:ab,ti OR ‘metampicillin’:ab,ti OR ‘meticillin’:ab,ti OR ‘mezlocillin’:ab,ti OR ‘miraxid’:ab,ti OR ‘nafcillin’:ab,ti OR ‘optocillin’:ab,ti OR ‘oxacillin’:ab,ti OR ‘penamecillin’:ab,ti OR ‘penethamate’:ab,ti OR ‘penicillic acid’:ab,ti OR ‘penicillin’:ab,ti OR ‘penicilloic acid’:ab,ti OR ‘pheneticillin’:ab,ti OR ‘piperacillin’:ab,ti OR ‘pivampicillin’:ab,ti OR ‘pivmecillinam’:ab,ti OR ‘propicillin’:ab,ti OR ‘quinacillin’:ab,ti OR ‘retacillin’:ab,ti OR ‘sulbenicillin’:ab,ti OR ‘talampicillin’:ab,ti OR ‘tameticillin’:ab,ti OR ‘temocillin’:ab,ti OR ‘ticarcillin’:ab,ti OR ‘tobicillin’:ab,ti OR ‘triplopen’:ab,ti OR ‘ureidopenicillin’:ab,ti OR ‘avibactam’:ab,ti OR ‘brobactam’:ab,ti OR ‘clavulanic acid’:ab,ti OR ‘nacubactam’:ab,ti OR ‘relebactam’:ab,ti OR ‘sulbactam’:ab,ti OR ‘tazobactam’:ab,ti OR ‘timentin’:ab,ti OR ‘vaborbactam’:ab,ti OR ‘zidebactam’:ab,ti OR ‘albomycin’:ab,ti OR ‘Amdinocillin’:ab,ti OR 'amifloxacin':ab,ti OR 'Amikacin':ab,ti OR 'antofloxacin':ab,ti OR ‘Apramycin’:ab,ti OR ‘Avilamycin’:ab,ti OR ‘Azithromycin’:ab,ti OR ‘Bacitracin’:ab,ti OR ‘Bacteriocins’:ab,ti OR ‘Balofloxacin’:ab,ti OR ‘bekanamycin’:ab,ti OR ‘benzathine benzylpenicillin’:ab,ti OR ‘benzathine cloxacillin’:ab,ti OR ‘benzofuroquinolinium’:ab,ti OR ‘beta-Lactams’:ab,ti OR ‘Cephaloridine’:ab,ti OR ‘Cephacetrile’:ab,ti OR ‘Cephalexin’:ab,ti OR ‘Cephaloglycin’:ab,ti OR ‘Cephalothin’:ab,ti OR ‘Cephapirin’:ab,ti OR ‘Cephradine’:ab,ti OR ‘Cethromycin’:ab,ti OR ‘Chlortetracycline’:ab,ti OR ‘Cilastatin’:ab,ti OR ‘Ciprofloxacin’:ab,ti OR ‘Clarithromycin’:ab,ti OR ‘Clinafloxacin’:ab,ti OR ‘Clindamycin’:ab,ti OR ‘Clofazimine’:ab,ti OR ‘Colistin’:ab,ti OR ‘Daptomycin’:ab,ti OR ‘Dibekacin’:ab,ti OR ‘Doxycycline’:ab,ti OR ‘Edeine’:ab,ti OR ‘Enoxacin’:ab,ti OR ‘Enrofloxacin’:ab,ti OR ‘Erythromycin’:ab,ti OR ‘Finafloxacin’:ab,ti OR ‘Floxacillin’:ab,ti OR ‘Fluoroquinolone*’:ab,ti OR ‘Fosfomycin’:ab,ti OR ‘Fosmidomycin’:ab,ti OR ‘Gemifloxacin’:ab,ti OR ‘Grepafloxaci’:ab,ti OR ‘Lactam*’:ab,ti OR ‘Levofloxacin’:ab,ti OR ‘Lincosamide*’:ab,ti OR ‘Lomefloxacin’:ab,ti OR ‘Marbofloxacin’:ab,ti OR ‘Methampicillin’:ab,ti OR ‘Methicillin’:ab,ti OR ‘Meropenem’:ab,ti OR ‘Moxalactam’:ab,ti OR ‘Moxifloxacin’:ab,ti OR ‘Mupirocin’:ab,ti OR ‘Nadifloxacin’:ab,ti OR ‘Nitrofurantoin’:ab,ti OR ‘Norfloxacin’:ab,ti OR ‘Nystatin’:ab,ti OR ‘Ofloxacin’:ab,ti OR ‘Oleandomycin’:ab,ti OR ‘Pefloxacin’:ab,ti OR ‘Penicillanic’:ab,ti OR ‘Penicillin*’:ab,ti OR ‘Pipemidic Acid’:ab,ti OR ‘Prulifloxacin’:ab,ti OR ‘Sparfloxacin’:ab,ti OR ‘Staphylococcin’:ab,ti OR ‘Sulfacetamide’:ab,ti OR ‘Sulfadiazine’:ab,ti OR ‘Sulfaguanol’:ab,ti OR ‘Sulfamerazine’:ab,ti OR ‘Sulfameter’:ab,ti OR ‘Sulfamethoxypyridazine’:ab,ti OR ‘Sulfanilamide’:ab,ti OR ‘Syringomycin’:ab,ti OR ‘Tedizolid’:ab,ti OR ‘Teicoplanin’:ab,ti OR ‘Temafloxacin’:ab,ti OR ‘Tetarimycin’:ab,ti OR ‘Tetracenomycin’:ab,ti OR ‘Tetracycline’:ab,ti OR ‘Tigecycline’:ab,ti OR ‘Tobramycin’:ab,ti OR ‘Tomaymycin’:ab,ti OR ‘Trimethoprim’:ab,ti OR ‘Sulfamethoxazole’:ab,ti OR ‘Ulifloxacin’:ab,ti OR ‘Vancomycin’:ab,ti OR 'doxorubicin':ab,ti OR 'adriamycin':ab,ti OR 'rufloxacin':ab,ti) AND (prevention:lnk OR 'prophylaxis'/de OR 'prevention'/de OR 'premedication'/de OR prevent*:ab,ti OR premedication:ab,ti OR premedications:ab,ti OR prophyla*:ab,ti)))

NOT

(('animal'/de OR 'animal experiment'/exp OR 'nonhuman'/de) NOT ('human'/exp OR 'human experiment'/de))

## Medline (Ovid)

(((Urologic Surgical Procedures/ OR (((transurethra* OR trans-urethra* OR intravesical OR vesical OR endovesical OR endoscopic OR outflow tract) ADJ3 (surger* OR resection* OR operation* OR procedure* OR surgical)) OR electroresection OR electro-resection OR cauterization).ab,ti.) AND (exp urinary bladder neoplasms/ OR Carcinoma, Transitional Cell/ OR exp Urinary Bladder Diseases/ OR urinary bladder/ OR urothelium/ OR bladder.ab,ti. OR vesica urinaria.ab,ti. OR intravesical.ab,ti. OR vesical.ab,ti. OR urotheli*.ab,ti. OR urinary tract epitheli*.ab,ti. OR uroepitheli*.ab,ti.)) OR TURBT.ab,ti. OR TURB.ab,ti.)

AND (exp Antibiotic Prophylaxis/ OR infection control/ OR ((Anti-Infective Agents/ OR Anti-Bacterial Agents/ OR beta-Lactamase Inhibitors/ OR Anti-Infective Agents, Urinary/ OR antibiotic.ab,ti. OR antibiotics.ab,ti. OR anti-biotic.ab,ti. OR anti-biotics.ab,ti. OR anti-bacterial.ab,ti. OR antibacterial.ab,ti. OR bacteriocidal.ab,ti. OR bacteriocide.ab,ti. OR bacteriocides.ab,ti. OR Antiinfective.ab,ti. OR Anti-infective.ab,ti. OR antiseptic.ab,ti. OR antiseptics.ab,ti. OR anti-septic.ab,ti. OR anti-septics.ab,ti. OR antimicrobial.ab,ti. OR anti-microbial.ab,ti. OR microbicide.ab,ti. OR microbicides.ab,ti. OR infection*.ab,ti. OR biapenem.ab,ti. OR brobactam.ab,ti. OR carbapenem.ab,ti. OR clavulanate potassium.ab,ti. OR clavulanic acid.ab,ti. OR doripenem.ab,ti. OR ertapenem.ab,ti. OR fropenem.ab,ti. OR imipenem.ab,ti. OR l 786392.ab,ti. OR lenapenem.ab,ti. OR meropenem.ab,ti. OR monobactam .ab,ti. OR nacubactam.ab,ti. OR nocardicin.ab,ti. OR nocardicinic acid .ab,ti. OR panipenem.ab,ti. OR pirazmonam.ab,ti. OR razupenem.ab,ti. OR ritipenem.ab,ti. OR sanfetrinem.ab,ti. OR sulbactam.ab,ti. OR sulopenem.ab,ti. OR sultamicillin.ab,ti. OR tazobactam.ab,ti. OR tebipenem.ab,ti. OR thienamycin.ab,ti. OR timentin.ab,ti. OR tomopenem.ab,ti. OR tribactam.ab,ti. OR trinem derivative.ab,ti. OR u 78608.ab,ti. OR benzathine cefalexin.ab,ti. OR benzathine cefapirin.ab,ti. OR carbacephem .ab,ti. OR cefacetrile.ab,ti. OR cefaclor.ab,ti. OR cefadroxil.ab,ti. OR cefalexin.ab,ti. OR cefaloglycin.ab,ti. OR cefaloram.ab,ti. OR cefaloridine.ab,ti. OR cefalotin.ab,ti. OR cefamandole.ab,ti. OR cefapirin.ab,ti. OR cefatrizine.ab,ti. OR cefazaflur.ab,ti. OR cefazedone.ab,ti. OR cefazolin.ab,ti. OR cefbuperazone.ab,ti. OR cefcanel.ab,ti. OR cefcapene.ab,ti. OR cefclidin.ab,ti. OR cefdaloxime.ab,ti. OR cefdinir.ab,ti. OR cefditoren.ab,ti. OR cefepime.ab,ti. OR cefetamet.ab,ti. OR cefetecol.ab,ti. OR cefiderocol.ab,ti. OR cefixime.ab,ti. OR cefluprenam.ab,ti. OR cefmatilen.ab,ti. OR cefmenoxime.ab,ti. OR cefmetazole.ab,ti. OR cefminox.ab,ti. OR cefodizime.ab,ti. OR cefonicid.ab,ti. OR cefoperazone.ab,ti. OR ceforanide.ab,ti. OR cefoselis.ab,ti. OR cefotaxime.ab,ti. OR cefotetan.ab,ti. OR cefotiam.ab,ti. OR cefovecin.ab,ti. OR cefoxitin.ab,ti. OR cefozopran.ab,ti. OR cefpimizole.ab,ti. OR cefpiramide.ab,ti. OR cefpirome.ab,ti. OR cefpodoxime.ab,ti. OR cefprozil.ab,ti. OR cefquinome.ab,ti. OR cefradine.ab,ti. OR cefroxadine.ab,ti. OR cefsulodin.ab,ti. OR ceftaroline.ab,ti. OR ceftazidime.ab,ti. OR cefteram.ab,ti. OR ceftezole.ab,ti. OR ceftibuten.ab,ti. OR ceftiofur.ab,ti. OR ceftizoxime.ab,ti. OR ceftobiprole.ab,ti. OR ceftolozane.ab,ti. OR ceftriaxone.ab,ti. OR cefuroxime.ab,ti. OR cefuzonam.ab,ti. OR cephalosporin*.ab,ti. OR cephamycin.ab,ti. OR deacetoxycephalosporin C.ab,ti. OR deacetylcefotaxime.ab,ti. OR deacetylcephalosporin C.ab,ti. OR fleroxacin deacetylcefotaxime ester.ab,ti. OR flomoxef.ab,ti. OR latamoxef.ab,ti. OR loracarbef.ab,ti. OR nitrocefin.ab,ti. OR thiophenoxycefalotin.ab,ti. OR aztreonam.ab,ti. OR carumonam.ab,ti. OR gloximonam.ab,ti. OR sulfazecin.ab,ti. OR tigemonam.ab,ti. OR adicillin.ab,ti. OR almecillin.ab,ti. OR aminopenicillin.ab,ti. OR amoxicillin.ab,ti. OR ampicillin.ab,ti. OR apalcillin.ab,ti. OR aspoxicillin.ab,ti. OR azidocillin.ab,ti. OR azlocillin.ab,ti. OR bacampicillin.ab,ti. OR bacmecillinam.ab,ti. OR carbenicillin.ab,ti. OR carfecillin.ab,ti. OR carindacillin.ab,ti. OR cloxacillin.ab,ti. OR cyclacillin.ab,ti. OR dicloxacillin.ab,ti. OR epicillin.ab,ti. OR flucloxacillin.ab,ti. OR flumoxil.ab,ti. OR fomidacillin.ab,ti. OR furbenicillin.ab,ti. OR fuzlocillin.ab,ti. OR hetacillin.ab,ti. OR isopenicillin N.ab,ti. OR lenampicillin.ab,ti. OR mecillinam.ab,ti. OR metampicillin.ab,ti. OR meticillin.ab,ti. OR mezlocillin.ab,ti. OR miraxid.ab,ti. OR nafcillin.ab,ti. OR optocillin.ab,ti. OR oxacillin.ab,ti. OR penamecillin.ab,ti. OR penethamate.ab,ti. OR penicillic acid.ab,ti. OR penicillin.ab,ti. OR penicilloic acid.ab,ti. OR pheneticillin.ab,ti. OR piperacillin.ab,ti. OR pivampicillin.ab,ti. OR pivmecillinam.ab,ti. OR propicillin.ab,ti. OR quinacillin.ab,ti. OR retacillin.ab,ti. OR sulbenicillin.ab,ti. OR talampicillin.ab,ti. OR tameticillin.ab,ti. OR temocillin.ab,ti. OR ticarcillin.ab,ti. OR tobicillin.ab,ti. OR triplopen.ab,ti. OR ureidopenicillin.ab,ti. OR avibactam.ab,ti. OR brobactam.ab,ti. OR clavulanic acid.ab,ti. OR nacubactam.ab,ti. OR relebactam.ab,ti. OR sulbactam.ab,ti. OR tazobactam.ab,ti. OR timentin.ab,ti. OR vaborbactam.ab,ti. OR zidebactam.ab,ti. OR albomycin.ab,ti. OR Amdinocillin.ab,ti. OR amifloxacin.ab,ti. OR Amikacin.ab,ti. OR antofloxacin.ab,ti. OR Apramycin.ab,ti. OR Avilamycin.ab,ti. OR Azithromycin.ab,ti. OR Bacitracin.ab,ti. OR Bacteriocins.ab,ti. OR Balofloxacin.ab,ti. OR bekanamycin.ab,ti. OR benzathine benzylpenicillin.ab,ti. OR benzathine cloxacillin.ab,ti. OR benzofuroquinolinium.ab,ti. OR beta-Lactams.ab,ti. OR Cephaloridine.ab,ti. OR Cephacetrile.ab,ti. OR Cephalexin.ab,ti. OR Cephaloglycin.ab,ti. OR Cephalothin.ab,ti. OR Cephapirin.ab,ti. OR Cephradine.ab,ti. OR Cethromycin.ab,ti. OR Chlortetracycline.ab,ti. OR Cilastatin.ab,ti. OR Ciprofloxacin.ab,ti. OR Clarithromycin.ab,ti. OR Clinafloxacin.ab,ti. OR Clindamycin.ab,ti. OR Clofazimine.ab,ti. OR Colistin.ab,ti. OR Daptomycin.ab,ti. OR Dibekacin.ab,ti. OR Doxycycline.ab,ti. OR Edeine.ab,ti. OR Enoxacin.ab,ti. OR Enrofloxacin.ab,ti. OR Erythromycin.ab,ti. OR Finafloxacin.ab,ti. OR Floxacillin.ab,ti. OR Fluoroquinolone*.ab,ti. OR Fosfomycin.ab,ti. OR Fosmidomycin.ab,ti. OR Gemifloxacin.ab,ti. OR Grepafloxaci.ab,ti. OR Lactam*.ab,ti. OR Levofloxacin.ab,ti. OR Lincosamide*.ab,ti. OR Lomefloxacin.ab,ti. OR Marbofloxacin.ab,ti. OR Methampicillin.ab,ti. OR Methicillin.ab,ti. OR Meropenem.ab,ti. OR Moxalactam.ab,ti. OR Moxifloxacin.ab,ti. OR Mupirocin.ab,ti. OR Nadifloxacin.ab,ti. OR Nitrofurantoin.ab,ti. OR Norfloxacin.ab,ti. OR Nystatin.ab,ti. OR Ofloxacin.ab,ti. OR Oleandomycin.ab,ti. OR Pefloxacin.ab,ti. OR Penicillanic.ab,ti. OR Penicillin*.ab,ti. OR Pipemidic Acid.ab,ti. OR Prulifloxacin.ab,ti. OR Sparfloxacin.ab,ti. OR Staphylococcin.ab,ti. OR Sulfacetamide.ab,ti. OR Sulfadiazine.ab,ti. OR Sulfaguanol.ab,ti. OR Sulfamerazine.ab,ti. OR Sulfameter.ab,ti. OR Sulfamethoxypyridazine.ab,ti. OR Sulfanilamide.ab,ti. OR Syringomycin.ab,ti. OR Tedizolid.ab,ti. OR Teicoplanin.ab,ti. OR Temafloxacin.ab,ti. OR Tetarimycin.ab,ti. OR Tetracenomycin.ab,ti. OR Tetracycline.ab,ti. OR Tigecycline.ab,ti. OR Tobramycin.ab,ti. OR Tomaymycin.ab,ti. OR Trimethoprim.ab,ti. OR Sulfamethoxazole.ab,ti. OR Ulifloxacin.ab,ti. OR Vancomycin.ab,ti. OR doxorubicin.ab,ti. OR adriamycin.ab,ti. OR rufloxacin.ab,ti.) AND ("prevention and control".fs. OR premedication/ OR prevent*.ab,ti. OR premedication.ab,ti. OR premedications.ab,ti. OR prophyla*.ab,ti.)))

NOT

(exp animals/ NOT humans/)

## CENTRAL

((((((transurethra* OR trans-urethra* OR intravesical OR vesical OR endovesical OR endoscopic OR ‘outflow tract’) NEAR/3 (surger* OR resection* OR operation* OR procedure* OR surgical)) OR electroresection OR electro-resection OR cauterization):ab,ti) AND (bladder:ab,ti OR ‘vesica urinaria’:ab,ti OR intravesical:ab,ti OR vesical:ab,ti OR urotheli*:ab,ti OR ‘urinary tract epitheli*’:ab,ti OR uroepitheli*:ab,ti)) OR TURBT:ab,ti OR TURB:ab,ti) AND (((antibiotic:ab,ti OR antibiotics:ab,ti OR anti-biotic:ab,ti OR anti-biotics:ab,ti OR anti-bacterial:ab,ti OR antibacterial:ab,ti OR bacteriocidal:ab,ti OR bacteriocide:ab,ti OR bacteriocides:ab,ti OR Antiinfective:ab,ti OR Anti-infective:ab,ti OR antiseptic:ab,ti OR antiseptics:ab,ti OR anti-septic:ab,ti OR anti-septics:ab,ti OR antimicrobial:ab,ti OR anti-microbial:ab,ti OR microbicide:ab,ti OR microbicides:ab,ti OR infection*:ab,ti OR ‘biapenem’:ab,ti OR ‘brobactam’:ab,ti OR ‘carbapenem’:ab,ti OR ‘clavulanate potassium’:ab,ti OR ‘clavulanic acid’:ab,ti OR ‘doripenem’:ab,ti OR ‘ertapenem’:ab,ti OR ‘fropenem’:ab,ti OR ‘imipenem’:ab,ti OR ‘l 786392’:ab,ti OR ‘lenapenem’:ab,ti OR ‘meropenem’:ab,ti OR ‘monobactam ‘:ab,ti OR ‘nacubactam’:ab,ti OR ‘nocardicin’:ab,ti OR ‘nocardicinic acid ‘:ab,ti OR ‘panipenem’:ab,ti OR ‘pirazmonam’:ab,ti OR ‘razupenem’:ab,ti OR ‘ritipenem’:ab,ti OR ‘sanfetrinem’:ab,ti OR ‘sulbactam’:ab,ti OR ‘sulopenem’:ab,ti OR ‘sultamicillin’:ab,ti OR ‘tazobactam’:ab,ti OR ‘tebipenem’:ab,ti OR ‘thienamycin’:ab,ti OR ‘timentin’:ab,ti OR ‘tomopenem’:ab,ti OR ‘tribactam’:ab,ti OR ‘trinem derivative’:ab,ti OR ‘u 78608’:ab,ti OR ‘benzathine cefalexin’:ab,ti OR ‘benzathine cefapirin’:ab,ti OR ‘carbacephem ‘:ab,ti OR ‘cefacetrile’:ab,ti OR ‘cefaclor’:ab,ti OR ‘cefadroxil’:ab,ti OR ‘cefalexin’:ab,ti OR ‘cefaloglycin’:ab,ti OR ‘cefaloram’:ab,ti OR ‘cefaloridine’:ab,ti OR ‘cefalotin’:ab,ti OR ‘cefamandole’:ab,ti OR ‘cefapirin’:ab,ti OR ‘cefatrizine’:ab,ti OR ‘cefazaflur’:ab,ti OR ‘cefazedone’:ab,ti OR ‘cefazolin’:ab,ti OR ‘cefbuperazone’:ab,ti OR ‘cefcanel’:ab,ti OR ‘cefcapene’:ab,ti OR ‘cefclidin’:ab,ti OR ‘cefdaloxime’:ab,ti OR ‘cefdinir’:ab,ti OR ‘cefditoren’:ab,ti OR ‘cefepime’:ab,ti OR ‘cefetamet’:ab,ti OR ‘cefetecol’:ab,ti OR ‘cefiderocol’:ab,ti OR ‘cefixime’:ab,ti OR ‘cefluprenam’:ab,ti OR ‘cefmatilen’:ab,ti OR ‘cefmenoxime’:ab,ti OR ‘cefmetazole’:ab,ti OR ‘cefminox’:ab,ti OR ‘cefodizime’:ab,ti OR ‘cefonicid’:ab,ti OR ‘cefoperazone’:ab,ti OR ‘ceforanide’:ab,ti OR ‘cefoselis’:ab,ti OR ‘cefotaxime’:ab,ti OR ‘cefotetan’:ab,ti OR ‘cefotiam’:ab,ti OR ‘cefovecin’:ab,ti OR ‘cefoxitin’:ab,ti OR ‘cefozopran’:ab,ti OR ‘cefpimizole’:ab,ti OR ‘cefpiramide’:ab,ti OR ‘cefpirome’:ab,ti OR ‘cefpodoxime’:ab,ti OR ‘cefprozil’:ab,ti OR ‘cefquinome’:ab,ti OR ‘cefradine’:ab,ti OR ‘cefroxadine’:ab,ti OR ‘cefsulodin’:ab,ti OR ‘ceftaroline’:ab,ti OR ‘ceftazidime’:ab,ti OR ‘cefteram’:ab,ti OR ‘ceftezole’:ab,ti OR ‘ceftibuten’:ab,ti OR ‘ceftiofur’:ab,ti OR ‘ceftizoxime’:ab,ti OR ‘ceftobiprole’:ab,ti OR ‘ceftolozane’:ab,ti OR ‘ceftriaxone’:ab,ti OR ‘cefuroxime’:ab,ti OR ‘cefuzonam’:ab,ti OR ‘cephalosporin*’:ab,ti OR ‘cephamycin’:ab,ti OR ‘deacetoxycephalosporin C’:ab,ti OR ‘deacetylcefotaxime’:ab,ti OR ‘deacetylcephalosporin C’:ab,ti OR ‘fleroxacin deacetylcefotaxime ester’:ab,ti OR ‘flomoxef’:ab,ti OR ‘latamoxef’:ab,ti OR ‘loracarbef’:ab,ti OR ‘nitrocefin’:ab,ti OR ‘thiophenoxycefalotin’:ab,ti OR ‘aztreonam’:ab,ti OR ‘carumonam’:ab,ti OR ‘gloximonam’:ab,ti OR ‘sulfazecin’:ab,ti OR ‘tigemonam’:ab,ti OR ‘adicillin’:ab,ti OR ‘almecillin’:ab,ti OR ‘aminopenicillin’:ab,ti OR ‘amoxicillin’:ab,ti OR ‘ampicillin’:ab,ti OR ‘apalcillin’:ab,ti OR ‘aspoxicillin’:ab,ti OR ‘azidocillin’:ab,ti OR ‘azlocillin’:ab,ti OR ‘bacampicillin’:ab,ti OR ‘bacmecillinam’:ab,ti OR ‘carbenicillin’:ab,ti OR ‘carfecillin’:ab,ti OR ‘carindacillin’:ab,ti OR ‘cloxacillin’:ab,ti OR ‘cyclacillin’:ab,ti OR ‘dicloxacillin’:ab,ti OR ‘epicillin’:ab,ti OR ‘flucloxacillin’:ab,ti OR ‘flumoxil’:ab,ti OR ‘fomidacillin’:ab,ti OR ‘furbenicillin’:ab,ti OR ‘fuzlocillin’:ab,ti OR ‘hetacillin’:ab,ti OR ‘isopenicillin N’:ab,ti OR ‘lenampicillin’:ab,ti OR ‘mecillinam’:ab,ti OR ‘metampicillin’:ab,ti OR ‘meticillin’:ab,ti OR ‘mezlocillin’:ab,ti OR ‘miraxid’:ab,ti OR ‘nafcillin’:ab,ti OR ‘optocillin’:ab,ti OR ‘oxacillin’:ab,ti OR ‘penamecillin’:ab,ti OR ‘penethamate’:ab,ti OR ‘penicillic acid’:ab,ti OR ‘penicillin’:ab,ti OR ‘penicilloic acid’:ab,ti OR ‘pheneticillin’:ab,ti OR ‘piperacillin’:ab,ti OR ‘pivampicillin’:ab,ti OR ‘pivmecillinam’:ab,ti OR ‘propicillin’:ab,ti OR ‘quinacillin’:ab,ti OR ‘retacillin’:ab,ti OR ‘sulbenicillin’:ab,ti OR ‘talampicillin’:ab,ti OR ‘tameticillin’:ab,ti OR ‘temocillin’:ab,ti OR ‘ticarcillin’:ab,ti OR ‘tobicillin’:ab,ti OR ‘triplopen’:ab,ti OR ‘ureidopenicillin’:ab,ti OR ‘avibactam’:ab,ti OR ‘brobactam’:ab,ti OR ‘clavulanic acid’:ab,ti OR ‘nacubactam’:ab,ti OR ‘relebactam’:ab,ti OR ‘sulbactam’:ab,ti OR ‘tazobactam’:ab,ti OR ‘timentin’:ab,ti OR ‘vaborbactam’:ab,ti OR ‘zidebactam’:ab,ti OR ‘albomycin’:ab,ti OR ‘Amdinocillin’:ab,ti OR 'amifloxacin':ab,ti OR 'Amikacin':ab,ti OR 'antofloxacin':ab,ti OR ‘Apramycin’:ab,ti OR ‘Avilamycin’:ab,ti OR ‘Azithromycin’:ab,ti OR ‘Bacitracin’:ab,ti OR ‘Bacteriocins’:ab,ti OR ‘Balofloxacin’:ab,ti OR ‘bekanamycin’:ab,ti OR ‘benzathine benzylpenicillin’:ab,ti OR ‘benzathine cloxacillin’:ab,ti OR ‘benzofuroquinolinium’:ab,ti OR ‘beta-Lactams’:ab,ti OR ‘Cephaloridine’:ab,ti OR ‘Cephacetrile’:ab,ti OR ‘Cephalexin’:ab,ti OR ‘Cephaloglycin’:ab,ti OR ‘Cephalothin’:ab,ti OR ‘Cephapirin’:ab,ti OR ‘Cephradine’:ab,ti OR ‘Cethromycin’:ab,ti OR ‘Chlortetracycline’:ab,ti OR ‘Cilastatin’:ab,ti OR ‘Ciprofloxacin’:ab,ti OR ‘Clarithromycin’:ab,ti OR ‘Clinafloxacin’:ab,ti OR ‘Clindamycin’:ab,ti OR ‘Clofazimine’:ab,ti OR ‘Colistin’:ab,ti OR ‘Daptomycin’:ab,ti OR ‘Dibekacin’:ab,ti OR ‘Doxycycline’:ab,ti OR ‘Edeine’:ab,ti OR ‘Enoxacin’:ab,ti OR ‘Enrofloxacin’:ab,ti OR ‘Erythromycin’:ab,ti OR ‘Finafloxacin’:ab,ti OR ‘Floxacillin’:ab,ti OR ‘Fluoroquinolone*’:ab,ti OR ‘Fosfomycin’:ab,ti OR ‘Fosmidomycin’:ab,ti OR ‘Gemifloxacin’:ab,ti OR ‘Grepafloxaci’:ab,ti OR ‘Lactam*’:ab,ti OR ‘Levofloxacin’:ab,ti OR ‘Lincosamide*’:ab,ti OR ‘Lomefloxacin’:ab,ti OR ‘Marbofloxacin’:ab,ti OR ‘Methampicillin’:ab,ti OR ‘Methicillin’:ab,ti OR ‘Meropenem’:ab,ti OR ‘Moxalactam’:ab,ti OR ‘Moxifloxacin’:ab,ti OR ‘Mupirocin’:ab,ti OR ‘Nadifloxacin’:ab,ti OR ‘Nitrofurantoin’:ab,ti OR ‘Norfloxacin’:ab,ti OR ‘Nystatin’:ab,ti OR ‘Ofloxacin’:ab,ti OR ‘Oleandomycin’:ab,ti OR ‘Pefloxacin’:ab,ti OR ‘Penicillanic’:ab,ti OR ‘Penicillin*’:ab,ti OR ‘Pipemidic Acid’:ab,ti OR ‘Prulifloxacin’:ab,ti OR ‘Sparfloxacin’:ab,ti OR ‘Staphylococcin’:ab,ti OR ‘Sulfacetamide’:ab,ti OR ‘Sulfadiazine’:ab,ti OR ‘Sulfaguanol’:ab,ti OR ‘Sulfamerazine’:ab,ti OR ‘Sulfameter’:ab,ti OR ‘Sulfamethoxypyridazine’:ab,ti OR ‘Sulfanilamide’:ab,ti OR ‘Syringomycin’:ab,ti OR ‘Tedizolid’:ab,ti OR ‘Teicoplanin’:ab,ti OR ‘Temafloxacin’:ab,ti OR ‘Tetarimycin’:ab,ti OR ‘Tetracenomycin’:ab,ti OR ‘Tetracycline’:ab,ti OR ‘Tigecycline’:ab,ti OR ‘Tobramycin’:ab,ti OR ‘Tomaymycin’:ab,ti OR ‘Trimethoprim’:ab,ti OR ‘Sulfamethoxazole’:ab,ti OR ‘Ulifloxacin’:ab,ti OR ‘Vancomycin’:ab,ti OR 'doxorubicin':ab,ti OR 'adriamycin':ab,ti OR 'rufloxacin':ab,ti) AND (prevent*:ab,ti OR premedication:ab,ti OR premedications:ab,ti OR prophyla*:ab,ti)))
