# Supplementary material for: Antibiotic prophylaxis in transurethral resection of bladder tumours: study protocol for a systematic review and meta-analysis
Source: Syst Rev. 2020 Apr 23;9:89. doi: 10.1186/s13643-020-01353-2 (PMC7181504; doi:10.1186/s13643-020-01353-2)
Supplement: Supplementary file 3 — Additional file 3. [file 13643_2020_1353_MOESM3_ESM.docx]

# Additional file 3: Search strategy for risk factors for UTIs after TURB

## Medline (Ovid)

(((Urologic Surgical Procedures/ OR (((transurethra* OR trans-urethra* OR intravesical OR vesical OR endovesical OR endoscopic OR outflow tract) ADJ3 (surger* OR resection* OR operation* OR procedure* OR surgical)) OR electroresection OR electro-resection OR cauterization).ab,ti.) AND (exp urinary bladder neoplasms/ OR Carcinoma, Transitional Cell/ OR exp Urinary Bladder Diseases/ OR urinary bladder/ OR urothelium/ OR bladder.ab,ti. OR vesica urinaria.ab,ti. OR intravesical.ab,ti. OR vesical.ab,ti. OR urotheli*.ab,ti. OR urinary tract epitheli*.ab,ti. OR uroepitheli*.ab,ti.)) OR TURBT.ab,ti. OR TURB.ab,ti.)

AND (exp Urinary Tract Infections/ OR exp Cystitis/ OR exp Pyelitis/ OR Urethritis/ OR Epididymitis/ OR Prostatitis/ OR (((Urinary Tract OR bladder OR intravesical OR vesical OR endovesical OR perivesical OR kidney* OR renal OR urethr* OR epididym* OR urotheli* OR uroepitheli* OR urogenital OR outflow tract OR ureter* OR prostate* OR genitourinary OR tractus urogenitalis OR urine OR urinary OR urologic) ADJ3 (Infection* OR infectous OR infected OR Inflammation* OR inflammatory OR postoperative OR post-operative OR contaminat* OR microbiology*)) OR UTI OR pyuria OR pyurias OR bacteriuria OR bacteriurias OR Cystitis OR megacystitis OR pericystitis OR Cystitides OR Painful Bladder Syndrome OR pyelocystitis OR cystopyelitis OR pyelitis OR pyelonephritis OR pyelonephritides OR Cystopyelitis OR Urethritis OR Urethritides OR Epididymitis OR epidydimitis OR urosepsis OR uroseptic OR pyelonephros* OR pyonephros* OR hydroureteronephrosis OR Prostatitis OR Prostatitides).ti,ab.)

NOT

(exp animals/ NOT humans/)
